# Sex-Dependent Differences in Colorectal Cancer: With a Focus on Obesity

**DOI:** 10.3390/cells11223688

**Published:** 2022-11-20

**Authors:** Prachi Wele, Xian Wu, Haifei Shi

**Affiliations:** 1 Department of Biology, Miami University, Oxford, OH 45056, USA; 2 Department of Kinesiology, Nutrition, and Health, Miami University, Oxford, OH 45056, USA

**Keywords:** colorectal cancer, sex hormone, estrogen, obesity, adipokine, inflammatory cytokine

## Abstract

Colorectal cancer (CRC) is the third most common cancer and has the second highest cancer-related mortality in the world. The incident rates of CRC vary country-wise; however, population studies and data from different countries show a general increase in the CRC rate in young adults, males, and females ≥65 years. CRC incidence is affected by age, sex, environmental, dietary, hormonal, and lifestyle factors. Obesity is a known disease that is spreading rapidly throughout the world. A large body of literature indicates that, among many conditions, obesity is the increasing cause of CRC. Even though obesity is one of the known factors for CRC development, limited studies are available that explain the mechanistic link between obesity, sex hormones, and CRC development. Thus, this review summarizes the literature and aims to understand sex-dependent differences in CRC, especially in the context of obesity.

## 1. Introduction

According to the latest 2020 count from the Global Cancer Observatory (GCO) by the World Health Organization (WHO), colorectal cancer (CRC) is the third most common cancer after breast cancer and lung cancer (male and female cases combined), the third most common cancer in males after lung cancer and prostate cancer, and the second most common cancer after lung cancer in females [1]. The GCO database by the WHO also enlisted CRC as the second most deadly cancer in the world after lung cancer [1], including in the US [2]. In 2020, 1.93 million cases of CRC and 0.9 million deaths from CRC were reported worldwide [3,4]. The global CRC incident rate is estimated to increase by 60% in 2030 [5], and the number of CRC cases is predicted to reach 3.2 million by 2040 [3]. CRC incidence varies geographically. The number of CRC cases in 2020 was highest in China followed by the United States and India [1]. In 2020, China alone held 28.8% of CRC cases worldwide [4]. Furthermore, countries in the West (e.g., the UK, the US, and Australia) and the East (e.g., Japan, China, Singapore, and India) are both at a high risk for CRC [5]. Collectively, it can be concluded that there is and will be an increase in the CRC burden worldwide, including in both developed and developing countries, making it crucial to address and better understand CRC.

CRC is developed gradually owing to several molecular, genetic, and epigenetic changes [6]. Although the etiology of cancer is rather complex and difficult to trace because of changes in multiple different pathways, few review studies have attempted to describe early events that are specific to CRC [6,7]. CRC starts with an aberrant crypt in the mucosal epithelial layer of the colon and/or rectum followed by the development of neoplastic polyps; the sustenance of this neoplastic polyp for over a period leads to tumorigenesis of CRC [7]. Multiple genetic and epigenetic changes control the regulation of molecular pathways namely chromosome instability (CIN), microsatellite instability (MSI), and the CpG island methylator phenotype (CIMP) that are specifically dysregulated in CRC [6]. CIN observed in CRC includes gain or loss of a whole arm of chromosome, oncogene stress-induced genomic instability, and telomere erosion [8]. Certain MSI markers such as BAT-25, BAT-26, NR-21, NR-24, and MONO-27 are specific to CRC and are used for identification and lack the DNA mismatch repair (MMR) system [9]. Individuals with Lynch syndrome and mutations in MMR genes such as *MLH1*, *MSH2*, *MSH6*, and *PMS2* develop MSI CRC in the course of time [10]. The CIMP is marked by the hypermethylation of CpG islands, global hypomethylation, and mutations in the chromosome remodeling gene *CHD8* [8]. Moreover, DNA methylation of *BMP3*, *NDRG4* [11], and *VIM* [12] as well as mutations in *KRAS* are used for the diagnosis of CRC [11]. Due to unclear and limited knowledge of the etiology of CRC, available effective prevention could be inept considering the complexity of CRC.

Approximately 7–10% of CRC cases are inherited, including hereditary non-polyposis colorectal cancer (HNPCC or Lynch syndrome), familial adenomatous polyposis (FAP), MUTYH-associated polyposis (MAP), hamartomatous polyps such as Peutz-Jeghers syndrome (PJS), juvenile polyposis syndrome (JPS), and PTEN hamartoma tumor syndrome (PHTS) [13]. Moreover, female CRC cases are more related to hypermethylation, MSI, CIMP, and *BRAF* and *KRAS* mutations than male CRC cases [14]. Non-MSI hypermutability, epigenetic instability, and aberrant DNA mutations are also positively associated with CRC incidence [8].

**Figure 1:**
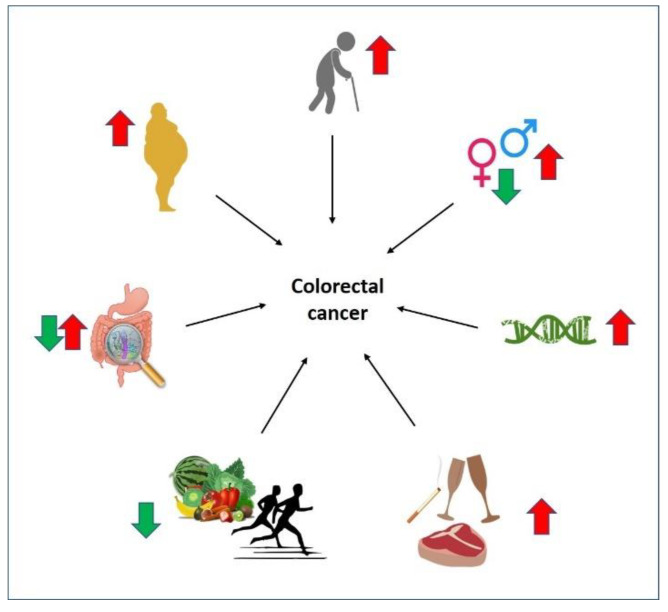
Factors influencing CRC risk. Factors influencing CRC risk are indicated by arrows where a red arrow indicates high risk while a green arrow low risk of developing CRC. (1) Elderly people are at high risk of CRC development; (2) compared to males, females have a lower risk of developing CRC due to the protective effect of estrogen. However, CRC development risk is increased in females over 65 years of age compared to males of similar age; (3) molecular changes such as CIN, MSI, and CIMP as well as families with a strong history of CRC increase the risk of developing CRC; (4) lifestyle of high consumption of alcohol, heavy smoking, and consumption of red meat and processed red meat amplifies CRC risk; (5) on the other hand, regular exercise and dietary habit of including fruits and vegetables that have high fibers have been reported to reduce CRC risk; (6) alteration of the gut microbiome can affect the CRC risk in both directions; (7) disease such as obesity has been strongly related to CRC development; moreover, obese males are at higher risk of developing CRC than obese females as females have the local estrogen production by adipose tissue.

Other factors—high consumption of alcohol, processed meat, sweetened beverages; low intake of fruits and vegetables; heavy smoking; and a sedentary lifestyle—increase the risk of CRC development [15] (Figure 1). Furthermore, obesity has been strongly associated with CRC development [16] (Figure 1). Other conditions such as inflammatory bowel diseases (IBD), including Crohn’s disease and ulcerative colitis, are also known to increase the CRC risk [17].

Interestingly, both CRC incidence and mortality are approximately 25% lower in females than in males [18]. An apparent explanation is that females are generally more willing to take screening tests for CRC; however, they are diagnosed with CRC at an advanced stage of stage II CRC while males are diagnosed at stage I CRC [14]. Although a global trend of males having higher rates of incidence and mortality for CRC has been noted, the rates are even higher for females over 65 years of age; additionally, the survival rate of females from young adult to middle age is higher than males, and this survival rate decreases for females ≥ 65 years compared to males [19]. These findings suggest a potential protective role of estrogen in CRC development. Furthermore, high consumption of meat and alcohol is also more prevalent in males than females which further adds a risk of developing CRC in males than in females [14]. Overall, there is a clear trend of sex-dependent differences in incidence, mortality, and survival rates of CRC. Despite that, only a few studies have addressed CRC through the lens of sex-related differences. Through this review, we aim to identify the sex-dependent differences and investigate underlying molecular mechanisms in CRC, with a particular focus on obesity. We acknowledge the non-binary spectrum of gender identities; hence, we unanimously used the phrase ‘sex-related, sex-dependent, or sex-based differences’ to address the dichotomy of CRC. 

## 2. Method

This is a narrative review of the current literature that centers around the role of sex-dependent differences in CRC, especially in the setting of obesity. We used various combinations of keywords, including colorectal cancer, CRC, pathogenesis, obesity, sex, gender, sex hormone, estrogen, sex difference, gender difference, males, and females in 3 databases, Google Scholar, PubMed, and ScienceDirect, with a year range of 2013–2022. Discussion of this review includes research articles, reviews, and reports published in English and considers epidemiological studies, population meta-analyses, animal studies, and clinical trials to understand CRC better. The present review consists of our experiential and explicit perspectives on this focused topic, which we feel is an essential first step toward developing therapies targeting sex-dependent factors in CRC prevention and treatment. Systematic reviews on pertinent topics using qualitative and quantitative methods are warranted to provide more valid evidence to guide clinical decision-making and pharmaceutical research and the development of new medicines.

## 3. Factors Influencing CRC

Several factors have been found to affect CRC incidence rates and mortality, including age, genetics, sex, location, lifestyle (such as lack of physical activity and smoking), diet and eating habits (high-calorie, fat-rich food, low-fiber food, and fewer vegetables and fruits), gut microbiome, and diseases and conditions such as obesity and IBD [20,21,22] (Figure 1). This section will provide a summary of major risk factors for CRC development.

### 3.1. Age

In the United States, the overall incidence rate of CRC is decreasing for people aged ≥50 years and is increasing for people aged <50 years (young adults) [23]. A similar pattern is observed among populations in The Netherlands [24], Denmark, New Zealand, Australia, the UK, Canada [25], Germany [26], Sweden, and Switzerland [27]. A few countries including Korea, Cyprus, India, Thailand, Turkey, Norway, Costa Rica, Finland, and Slovakia are showing an overall increased trend for CRC in both young adults and people aged ≥50 years [27]. On the other hand, an age-specific sex difference study in the US population showed that males have a higher incidence rate for CRC than females throughout their life, and females have a better survival rate only until the age of 64 years for colon cancer and 74 years for rectal cancer [28]. Furthermore, the equivalent level of CRC incidence and CRC-related mortality observed in males aged 50, 55, and 60 years is reached about 4–8 years later in females [29]. Thus, it can be considered that the influence of age on the incidence of CRC cases varies according to country, and there is no universal trend in CRC incidence. More studies are required to understand age-specific sex differences in CRC in different populations.

### 3.2. Sex

There is a difference in the survival rate from CRC depending on the sex of the patient. In general, the incidence of CRC is higher in males than that in females [13]. Females aged ≥65 years show higher mortality and a lower 5-year-survival compared to males of the same age [19]. In the US, the CRC incidence in females aged ≥65 years is 30 times more than that of young female adults [29]. Moreover, there is a difference in the CRC manifestation site depending on sex. In the majority of CRC cases, females have cancer tissue in the proximal colon—on the right side of the colon—while males have it in the distal colon—on the left side of the colon [19,30]. Due to the difficulty in screening the right side of the colon, the standard CRC screening methods such as sigmoidoscopy, colonoscopy, and the immunochemical fecal occult blood test (iFOBT) have less sensitivity in female patients compared to male patients [19]. Thus, this acts as a challenge as it may lead to screening of CRC in females being undetected and calls for screening updates. Additionally, males are more likely to develop metastatic colon cancer, and females are more likely to develop metastatic rectal cancer as they age [28]. Furthermore, the commonly used drugs for CRC patients showed sex-dependent differences in the drug response [31]. Pharmacological studies have found that females have a lower elimination rate than males due to sex-related differences in renal function; therefore, commonly used anti-cancer drugs can be toxic as they remain in the females’ bodies for a longer time [32]. This necessitates considering sex-dependent differences in drug responses in cancer patients.

### 3.3. Location, Lifestyle, and Diet

The incidence rate of CRC is high in developed countries and is alarmingly increasing in developing countries [33]. The number of CRC cases is highest in China, the US, and India [33]. Upon migration from CRC low-incident countries to CRC high-incident developed countries, immigrated people and the succeeding children are more vulnerable to developing CRC owing to changes in their lifestyles and diets [34]. Many studies argue that a Westernized lifestyle, including a sedentary lifestyle of low levels of physical activity along with high consumption of processed, high-calorie, fat-rich foods that supply excess energy, red meat, fiber-deficient food, alcohol, and tobacco, increases the CRC incidence rate [13,35,36]. Overall, location determines lifestyle and habits, and changes in the location, for example, migration from developing to developed countries, affect the lifestyle and food habits which themselves influence CRC risk. Thus, acclimating to a healthy lifestyle and diet is highly recommended as it might reduce the risk of CRC [5]. Sex-dependent differences in lifestyles have been documented where males tend to have more meat, alcohol, and heavy smoking; fewer fruits and vegetables; and a sedentary lifestyle compared to females [37]. These differences in habits may suggest the observed differences in the CRC incidence rate.

### 3.4. Gut Microbiome

The importance of the gut microbiome in the occurrence of CRC was recognized recently. Microbes weigh up to 1.5 kg in the human body and have been reported to contribute to the incidence and inflammation related to CRC [21]. The same study by Tomkovich et al. [21] found that germ-free Apc^Min/+^ and IL10^−/−^ 129/SvEv mice developed fewer tumors compared to gnotobiotic and specific-pathogen–free controls. A few review studies mentioned that *Escherichia coli* and *Bacteroides fragilis* promoted CRC carcinogenesis while *Akkermansia muciniphila* and *Faecalibacterium prausnitzii* prevented it [16,35]. Furthermore, the concentration of circulating lipopolysaccharide (LPS) was higher in a high-fat diet (HFD)-induced obesity owing to the remodeling of the gut microbiome [16]. This remodeling of the gut microbiome in CRC patients was shown to have an increased load of *Bacteroides*, *Fusobacterium*, *Dorea*, and *Porphyromonas*, and a reduced load of *Pseudomonas*, *Prevotella*, *Acinetobacter*, and *Catenibacterium* when compared with the bacterial analysis of normal healthy people [38]. Another independent study also found abundant *Fusobacterium nucleatum* in the tumor tissue sample and fecal matter of CRC patients [35]. Additionally, probiotics having specific strains of lactobacilli and bifidobacterial are reported to play a protective role against CRC [35]. One study recommended the use of probiotics along with conventional anti-cancer drugs as it improves gut conditions and produces anti-cancer compounds [35]. A recent study by Liao et al. [39] reported the presence of sex-dependent differences in the microbial community of the gut during the development of CRC in male and female CRC patients where the microbial community was more stable in males than in females. Sex hormones such as estrogen influence the gut microbiome; when male mice treated with azoxymethane/dextran sulfate sodium (AOM/DSS) were given estrogen (E2) treatment, the ratio of Firmicutes/Bacteroidetes was decreased, thereby reducing the risk of CRC in male mice [40]. Collectively, even though the importance of the gut microbiome in the occurrence of CRC has been recognized, only a few studies have addressed this; thus, more studies are essential.

### 3.5. Other Diseases Leading to CRC

Certain disorders increase CRC incidence and risk of developing CRC. IBD including Crohn’s disease and ulcerative colitis have been positively associated with increased CRC risk [17]. Another disease such as obesity is also reported to develop CRC in males and females [41]. Studies address the measurement of obesity by either body mass index (BMI), waist circumference (WC), or the waist-to-hip ratio (WHR). The WHO classifies a BMI over 25 kg/m^2^ as overweight and above 30 kg/m^2^ as obese. The impact of obesity on the occurrence of CRC is generally higher in males than in females due to the protective effect of female sex hormones such as estrogen [42]. Furthermore, the timing of estrogen administration is also important where early administration during the initiation of CRC development is the most effective period for the suppression of tumor development [43] and colon inflammation [44]. Short-chained fatty acids produced by the gut microbiome also act as a beneficial link as they can prevent obesity [16], which might offer a protective role against CRC. Thus obesity-induced CRC has sex-based differences due to the protective effect of sex hormones.

## 4. Sex-Dependent Differences in Obesity-Associated CRC

The relationship between obesity and CRC, and how obesity plays important roles in CRC development, was reviewed by many studies. Our focus is on sex-dependent differences in CRC. This review centers around sex-dependent differences in obesity-induced CRC (Table 1).

### 4.1. Obesity Increases CRC Incidence Differently in Males and Females

Many clinical and epidemiological studies demonstrate that obesity increases CRC risk and incidence [52]. However, even though the prevalence of obesity is higher in females than in males [16,47], obese males are at a higher risk of developing CRC than obese females [41]. A study on the European population was one of the initial meta-analysis studies to find a positive association between obesity and the occurrence of CRC where obese people had a 33% higher chance of developing CRC than normal people [53]. Following that, various studies chose to examine obesity either by measuring general obesity (BMI) or abdominal obesity (WC or WHR). A population study in Korea reported that people with a higher WC are more prone to colorectal, colon, and rectal cancer, and this association is stronger in males than females and is higher in old females aged ≥70 years than young females [54]. Another population study in China measured both general and abdominal obesity and found the same result of a positive association between obesity, both overall and abdominal, and CRC where the CRC risk is more in males than females [41].

A population cohort study in France reported that obese patients who underwent bariatric surgeries have a reduced risk of developing CRC which is the same as the general population aged 50–74 years [55]. The French population study also reported that the same age group of obese patients who did not undergo bariatric surgery have a 34% higher risk of developing CRC compared to patients who have undergone bariatric surgery. A similar observation of the reduced risk of developing CRC after bariatric surgery was reported in the population studies of Italy [56], England [57], and the US [58,59]. Furthermore, a meta-analysis study by an Australian research team found that obesity in young adults, especially increased weight change at a young age from early adulthood to midlife, is associated with a higher risk of CRC than those who develop obesity at an older age from midlife to old age [60].

### 4.2. Sex-Based Effect of Adipokines in Obesity-Associated CRC

The molecular mechanisms by which obesity increases the risk of developing CRC are not completely known due to the complexity involving mitogenic effects of various adipokines on stimulating proliferation and inhibiting apoptosis of tumor cells. Certain common features of adiposity that are observed in obesity-induced CRC patients are the increased presence of free fatty acids, lipids, and cholesterol levels; moreover, the increased concentration of insulin in the serum is also associated with increased CRC risk [34].

Adipose tissue acts as an endocrine tissue and releases many adipokine hormones, many of which are pro-inflammatory cytokines promoting cancer progressions, such as resistin, tumor necrosis factor α, visfatin, and interleukin 6 [47,61]. Two main adipokines are adiponectin and leptin. High levels of leptin and low levels of adiponectin in the serum are associated with obesity and act as a risk for the development of CRC [49]. Leptin acts on its receptor to stimulate the proliferation of colon epithelial cells and promote CRC progression [62] and has pro-inflammatory, mitogenic, and angiogenic properties [63]. On the contrary, adiponectin aids in apoptosis and inhibits the growth of tumors [49]. Furthermore, two single-nucleotide polymorphisms (SNPs) of the leptin gene, namely *LEP* rs2167270 and *LEP* rs4731426, only affect females by increasing their risk for CRC independent of obesity; the SNP variant of adiponectin *ADIPOQ* rs17366743 is positively associated with obesity only in males and thus poses a risk for the development of CRC [49]. Another study reported no association between *LEP* rs7799039 gene variants and CRC risk [64]. 

One clinical study in North Sweden CRC patients reported that the leptin level, but not BMI or the insulin level, is associated with CRC risk in men, but no association between leptin and CRC risk was found in women [65]. This study suggests that the obesity-elevated leptin level is associated with a higher risk of developing CRC in males. A few publications, however, suggest no link between the circulating leptin level and CRC risk in either men or women [63,64,66,67]. A meta-analysis study found no significant association between circulating leptin and CRC risk, while a higher level of adiponectin is significantly associated with a decreased CRC risk in men and all normal-weight people, but an increased risk among overweight people [66]. Another meta-analysis study suggests a positive association of serum leptin with colorectal adenoma but not with CRC risk [67]. Albeit sex-based differences in obesity-induced CRC risk and survival have been reported, not many studies have addressed the sex-dependent effects of adipokines on CRC.

### 4.3. Obesity-Induced Chronic Inflammation Sex-Dependently Affects CRC

Obesity also causes low-grade chronic inflammation and macrophage infiltration in the adipose tissue [68]. These macrophages secrete inflammatory cytokines, including interleukin 6 (IL-6), tumor necrosis factor-alpha (TNF-α), and monocyte chemotactic protein 1 (MCP-1) [16]. Studies have observed higher levels of IL-6 with an increased tumor stage of CRC [16,34]. Chronic inflammation has a positive effect on the development and progression of CRC and is marked by increased serum levels of C-reactive protein (CRP-1), TNF-α, and IL-6 [69]. The CRP-1 protein expressed by hepatocytes in the liver is a well-established marker for inflammation in CRC patients [70]. An elevated blood level of CRP-1 is strongly associated with CRC, and the association is stronger in males than in females [50]. In conclusion, fat deposition risks health; therefore, maintaining the BMI and/or WC or WHR within the normal range is highly recommended.

### 4.4. Obesity and Sex Hormones Affect CRC

#### 4.4.1. Estrogens and Their Receptors

Adipose tissue expresses estrogen in both males and females, while its relation is inverse with testosterone levels in males [71]. The estrogens found in the body are estrone (E1), 17-β estradiol (E2), and estriol (E3). Estrogen can bind to classical nuclear receptors, either estrogen receptor alpha (ESR1 or ER-α) and/or estrogen receptor beta (ESR2 or ER-β), and carry out genomic effects by having antagonistic consequences [72]. Upon binding to ER-α, the signaling stimulates proliferative signals via ERK/MAPK and PI3K/AKT pathways, while binding to ER-β activates anti-proliferative signals via phosphorylated p38/MAPK, caspase 3 activation, and cleavage of PARP [73]. Estrogen-bound estrogen receptors enter the nucleus and bind to the estrogen-responsive element (ERE) present in the promoter region of various genes [74]. Thus, a physical interaction between estrogen-bound estrogen receptors and ERE leads to the expression of estrogen-responsive genes. The other non-genomic mechanism is the binding of ER to transcription factors such as Sp-1, Ap-1, and NF-κB [73,74]. Estrogen can also bind to nonclassical membrane receptors present on the plasma membrane or nuclear membrane. Both ERs are also reported to interact with GPCR proteins, tyrosine kinase, and scaffolding proteins [30,75].

Sex hormones specific to their sexes have been reported to play a significant role in reducing CRC risk [71]. The effect of estrogen is highly studied as it reduces the risk of obesity-induced CRC in females [42]. The effect of leptin variants namely *LEP* rs2167270 and *LEP* rs4731426 that increased CRC risk independent of obesity in females is reduced by estrogen treatment [49]. Furthermore, in postmenopausal women, the risk of CRC occurrence is low if they have estrogen-only (EO) HRT but not the estrogen-progesterone therapy [49,76]. Additionally, never users of HRT were at more risk of CRC if they had the listed leptin variants [49]. The healthy colon tissues have a higher expression of ERβ than ERα; however, this ratio changes as the tumor progresses in CRC patients [30]. When wild-type C57BL/6J mice were treated with AOM/DSS and later with estrogen, estrogen reduced the multiplicity of tumors [44]. Similarly, when ovariectomized Min/+ mice were given estrogen treatment, the mice had lower ERα expression and higher ERβ in their enterocytes [74,77]. Thus, estrogen treatment incurs a protective effect by changing the ratio of ERβ/Erα [44].

#### 4.4.2. Androgens and Their Receptors

Androgen or testosterone binds to androgen receptors (ARs) in the colon and to the NR3C4 receptor in the nucleus which is a DNA-binding transcription factor that regulates the expression of many genes [78]. A population study in the US found that lower levels of androgen and sex hormone–binding globulin (SHBG) along with a lower estrogen-to-testosterone ratio are associated with a higher risk of CRC in males [71]. However, contradictory results were found in animal models, for example, orchidectomy Apc (Pirc/+) rats were comparatively more protected than those treated with testosterone supplementation [78,79].

When C57BL/6J mice were fed with a high-fat diet (HFD), males had a shortened colon, colon inflammation, and increased levels of blood glucose after 6 h fasting and an increased insulin level after 2 h fasting compared to their female counterparts [42]. Additionally, obesity lowers testosterone levels which further adds to the risk of developing CRC [80] due to the high activity of the aromatase enzyme by the adipose tissue that converts androgen to estrogen [78]. Another independent study by Yang et al. [81] found a positive relationship between high circulating levels of testosterone and low CRC risk. Thus, inadequate studies are available to understand the relationship between obesity, testosterone, and CRC; however, it is evident that males are more affected by diet-induced obesity than females with a higher probability of developing CRC.

## 5. Lifestyle and Its Sex-Dependent Impact on CRC

Consumption of alcohol and tobacco smoking are also associated with CRC risk. Alcohol consumption varies by sex where males frequently consume more alcohol compared to females; however, the difference in alcohol consumption between the sexes is decreasing [82]. A population study in the Netherlands found that females were at CRC risk when they were heavy drinkers (>30 g/day) while male drinkers were at risk if they were light and moderate drinkers (0.1–29 g/day) [45]. Moreover, gene variants of *ADH* have sex-dependent effects where the risk of distal CRC increases only in male drinkers having *ADH1B* rs4147536 SNP in the *ADH1B* gene, and the risk of proximal CRC increases in female drinkers having *ADH1C* rs283415 SNP in the *ADH1C* gene, whereas gene variant *ALDH2* rs671 in the *ALDH* gene slows the oxidation of acetaldehyde to acetate with no reported sex-dependent effects [45]. Nonetheless, not many population and meta-analysis studies have reported the sex-dependent effects of alcohol and/or tobacco and their association with CRC development thereafter. 

A review by Conti et al. [37] mentioned that females tend to have more healthy dietary habits than males, and females are more concerned about their diet, such as eating food with low calories, more consumption of fruits and vegetables, less alcohol consumption, and eating food rich in fiber, than males whose intake is pleasure-oriented. The same study also mentioned that these sex differences in dietary preference and habits are attributed to psychological and socio-cultural factors. These differences in attributes toward food and lifestyle might add to the reduced risk of CRC in females compared to males. Nonetheless, more population studies are required to discern the environmental, societal, cultural, and gender factors that might contribute to CRC development. 

## 6. Discussion

The global burden of CRC is estimated to increase in the future. There is an increased CRC rate in the young population, and CRC being the second-most deadly cancer in the world, it demands attention for additional research. The etiology of CRC includes the formation of polyps and polyps turning into tumors [14]. Molecular etiology includes changes in MSI, the CIMP, and CIN [6]. CRC also shows many sex-dependent variations including the incidence and mortality rates. The incidence rate of CRC is higher in males than in females. Moreover, obese males are at a higher risk of CRC than obese females. Furthermore, females ≥65 years have higher mortality than young females [27]. 

Given the fact that there are sex-related differences in CRC, including differences in the CRC incidence rate, CRC site, sensitivity in screening, hormonal effect, age, sex, tobacco smoking, dietary habits, and fat deposition, very few studies have addressed CRC considering these differences. For example, globally in 2020, 32.6% of men but only 6.5% of women were active smokers [46]. This difference might also contribute to the sex difference in CRC incidence. Another example is that CRC cases in females are diagnosed as emergency cases whereas male CRC cases are detected at an earlier stage [14]. This difference could be due to the less sensitivity of standard tests for female CRC patients [19]. Additionally, mutations related to CRC are sex-specific where mutations in *BRAF*, *KRAS*, *PIK3CA*, and *PTEN* are on the proximal colon while those in *APC* and *TP53* are on the distal colon [51]. 

The disparity in obesity assessment, diagnostic methods, and hormone measurement also makes CRC difficult to understand. For example, a few population studies conducted in Korea, China, and Taiwan found that abdominal obesity (with increased WC or WHR) was linked to CRC, while other population studies conducted in the US, The Netherlands, and Germany found that general obesity (with increased BMI) was linked to CRC. Overall, abdominal adiposity has been found to be more closely associated with CRC risk than general obesity [83]. Sexual dimorphism in the fat distribution has also been noted where premenopausal females have fat distributed on the thighs, hips, and buttocks while males have them in the abdominal region [48]. For fat deposition, females tend to have subcutaneous adipose tissue while males have visceral adipose tissue [47]. Additionally, Asian people have higher visceral fat than overall obesity while Caucasians have less visceral fat and more overall obesity for the same BMI [84]. Moreover, BMI cannot distinguish between fat mass and lean mass and does not consider fat distribution [85]. Thus, differences in fat distribution in Eastern and Western hemisphere populations owing to their food, lifestyle, habits, and socio-cultural status open a discussion topic on how to elucidate a universal trend between adiposity and CRC risk. One suggestion would be to gather information about the general as well as abdominal fat of CRC patients along with patients’ location, lifestyle, and habits in a global database. This might give a vivid picture of different types of obesity with region-specific fat distribution and its link to CRC.

Obese males have a 50–70% higher risk of developing CRC than normal males, while obese females have a 10–25% higher risk of developing CRC than normal females [34]. Obesity-induced CRC in C57BL/6J mice shows sex-dependent differences in the colon transcriptome. A differential expression of genes (DEG) related to cell cycles and hypoxia was found in obese male mice while a DEG related to lipid metabolism, steroid hormones, and Wnt signaling was reported in obese female mice [42]. Besides, the leading cause of death in both male and female obese CRC patients is cardiovascular disease [86]. Therefore, obesity not only affects the colon and/or rectum but also the physiology of other organs.

A meta-analysis study by Bouras et al. [87] reported no association between the endogenous concentration of sex hormones and CRC risk. Many studies have found that HRT treatment reduces CRC risk in females. An investigation of ovariectomy female ICR mice treated with AOM/DSS found that tumorigenesis is decreased when mice are provided with 17β-estradiol treatment [88]. A study by Chun et al. [49] also reported that estrogen-only therapy is better than combined estrogen-progesterone therapy. A similar observation was noted in post-menopausal women from the ‘Women Health Initiative’ project where conjugated equine estrogens therapy (CEE) had fewer risks than CEE plus medroxyprogesterone acetate (MPA) therapy (CEE + MPA); however, CEE or CEE + MPA groups had more severe effects such as stroke, pulmonary embolism, gallbladder disease, dementia, and urinary incontinence than the placebo group [76]. Unfortunately, tumor detection in post-menopausal CRC patients who are under combined HRT is at advanced stages of CRC, which reduces their prognosis [44]. Thus, HRT treatment in females might be delaying the occurrence of CRC, and the detection of CRC at advanced stages highly reduces survival chances. 

Some of the common features between the sexes of obesity-induced CRC include increased levels of leptin, IL-6, insulin, and C-reactive proteins (CRP-1), and decreased adiponectin levels [69,86]. Obesity is also shown to alter expression levels of genes involved in cell adhesion, cell proliferation, migration, angiogenesis, and immunity when checked in the colon epithelial cells of C57BL/6J mice in both sexes [42].

## 7. Conclusions

In conclusion, CRC is one of the cancers whose incidence is modifiable, and progression can be slowed by following healthy lifestyles and maintaining a BMI within a normal range such as regular exercise, healthy diets, and minimizing consumption of alcohol and tobacco. Sex-dependent lifestyles exist, including males having more physical activities than females throughout their lifetime, males’ diets rich in protein while females’ diets being rich in fiber, and more smoking and drinking in males compared to females. Even though sex-dependent differences in CRC are a notable phenomenon, only a few studies have addressed CRC through this point. Above that, studies on sex-dependent differences in obesity-induced CRC are even fewer. It is important to understand the influence of obesity and its sex-dependent effect on CRC. Advances in the understanding of CRC risks, pathophysiology, and underlying mechanisms would enhance the development of new treatment options and lead to the creation of individual treatment plans that are not the same for male and female patients.

## Figures and Tables

**Table 1:** Countable factors that have different effects on CRC in male and female CRC patients.

Factors of Differences in CRC	Male	Female
Incidence rate	High throughout life [28]	High at ≥65 years [19]
Mortality	More [18]	Less [18]
Willingness for screening tests	Less [14]	More [14]
CRC diagnosis stage	Stage I [14]	Stage II [14]
Survival advantage	High risk throughout life [28]	Low risk until 64 years for colon cancer and 74 years for rectal cancer [28]
Site of CRC	Distal colon [30]	Proximal colon [30]
Metastatic CRC	More likely to develop metastatic colon cancer [28]	More likely to develop metastatic rectal cancer [28]
Toxicity of commonly used anti-cancer drugs	Lower [31]	Higher [31]
Fiber intake	Lower [37]	Higher [37]
Sedentary lifestyle	Lower [37]	Higher [37]
Meat intake	Higher [14]	Lower [14]
Alcohol intake	Higher [14]	Lower [14]
Amount of alcohol consumption increases CRC risk	0.1–29 g/day [45]	>30 g/day [45]
Prevalence of smoking	Higher [46]	Lower [46]
Prevalence of obesity	Lower [47]	Higher [47]
Obesity-induced CRC risk	Higher [42]	Lower [42]
Fat distribution	Abdominal region [48]	Thighs, hips, and buttocks [48]
Fat deposition	Visceral adipose tissue [47]	Subcutaneous adipose tissue [47]
Leptin SNPs *LEP* rs2167270 and *LEP* rs4731426	No effect [49]	Increases risk, independent of obesity, only in females [49]
Adiponectin SNP *ADIPOQ* rs17366743	Increases risk of obesity in males and thus CRC risk [49]	No effect [49]
Risk of CRC when CRP-1 levels are elevated	Higher [50]	Lower [50]
Gene variants of alcohol dehydrogenase (ADH) enzyme increase cancer risk	*ADH1B* rs4147536 SNP increases the risk of distal colon cancer [45]	*ADH1C* rs283415 SNP increases the risk of proximal colon cancer [45]
Microbial community in the gut	More stable [39]	Less stable [39]
Mutations/epigenetics	Mutations in *APC* and *TP53* [51]	Hypermethylation, MSI, and CIMP, and mutations in *BRAF* and *KRAS* [14]

A combination of various keywords was used to study sex-dependent differences in CRC. Countable factors with different effects based on the sex of CRC patients are listed in the table.

## Data Availability

Not applicable.
